# From gaze cueing to perspective taking: Revisiting the claim that we automatically compute where or what other people are looking at

**DOI:** 10.1080/13506285.2015.1132804

**Published:** 2016-01-24

**Authors:** Henryk Bukowski, Jari K. Hietanen, Dana Samson

**Affiliations:** ^a^ Psychological Sciences Research Institute, Université catholique de Louvain, Louvain-La-Neuve, Belgium; ^b^ Social, Cognitive and Affective Neuroscience Unit, Department of Basic Psychological Research and Research Methods, Faculty of Psychology, University of Vienna, Vienna, Austria; ^c^ School of Social Sciences and Humanities/Psychology, Human Information Processing Laboratory, University of Tampere, Tampere, Finland

**Keywords:** Gaze, perspective-taking, attentional cueing, joint attention, theory of mind

## Abstract

Two paradigms have shown that people automatically compute what or where another person is looking at. In the visual perspective-taking paradigm, participants judge how many objects they see; whereas, in the gaze cueing paradigm, participants identify a target. Unlike in the former task, in the latter task, the influence of what or where the other person is looking at is only observed when the other person is presented alone before the task-relevant objects. We show that this discrepancy across the two paradigms is not due to differences in visual settings (Experiment 1) or available time to extract the directional information (Experiment 2), but that it is caused by how attention is deployed in response to task instructions (Experiment 3). Thus, the mere presence of another person in the field of view is not sufficient to compute where/what that person is looking at, which qualifies the claimed automaticity of such computations.

Our ability to compute what people see, known as visual perspective-taking (VPT), offers us very useful information during our social interactions (Baron-Cohen, 1995); it helps in understanding what other people talk about, what they like or dislike, what they intend to do, and what knowledge or beliefs of the world they form. Thus, VPT is an essential building block of “Theory of Mind”, that is, our ability to reason about other people's mental states (Premack & Woodruff, 1978).

The most basic form of VPT, also referred to as level 1 VPT, allows us to infer *what* someone else can or cannot see (as opposed to level 2 VPT which allows us to infer that an object may have a different appearance to someone else; Flavell, Everett, Croft, & Flavell, 1981). Level 1 VPT is known to be an early developing ability in children (Flavell et al., 1981) and is available even to nonhuman species (e.g., Hare, Call, Agnetta, & Tomasello, 2000). It has also been proposed that level 1 VPT is achieved via the computation of the other person's line of sight (Kessler & Rutherford, 2010; Michelon & Zacks, 2006; Surtees, Apperly, & Samson, 2013) and that such computation occurs automatically (Samson et al., 2010). For example, Samson et al. (2010) showed participants pictures of a room with discs pinned on the left and/or right wall and a centrally positioned human avatar facing either the left or the right wall (see Figure 1). The discs were displayed in such a way that, on half of the trials, some of these discs were not visible to the avatar. The authors found that when the participants were asked to verify the number of discs they could see from their own point of view, they were slowed down and less accurate in their judgments when the number of discs in the room did not match the number of discs that the avatar could see, suggesting that participants automatically processed *what* the avatar could see even when it was not relevant for their judgment.

**Figure 1.**
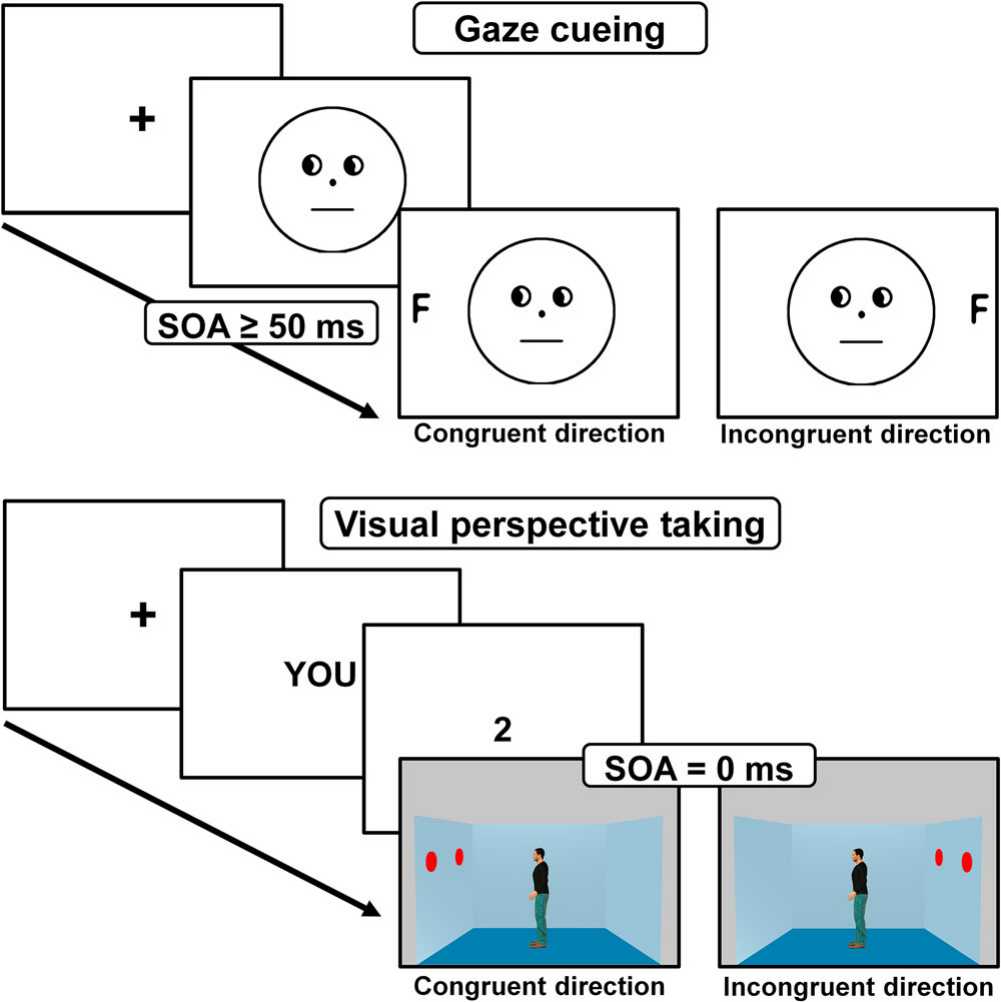
Upper panel: Illustration of the gaze cueing paradigm. A gazing face is first presented alone (SOA ≥ 50 ms) followed by the presentation of a target (often a letter to identify) at a location that is either congruent or incongruent with the gaze cue. Lower panel: Illustration of the level-1 visual perspective-taking paradigm. Two prompts indicate the perspective to take (here “YOU” instructs to take the self-perspective) and the perspective content to verify (here “2” refers to the number of discs) followed by the presentation of a scene in which another person is presented simultaneously (SOA = 0 ms) with the targets; the other person can either see the same or a different number of discs than participants.

Interestingly, in a parallel line of research it has been shown that our attention is reflexively oriented towards *where* someone else is looking. This phenomenon is typically observed in a gaze cueing paradigm where participants are faster at detecting or identifying an object when it appears in the gaze direction of a preceding social cue (e.g., eye gaze or head orientation) than when it appears in the opposite direction (Driver et al., 1999; Friesen & Kingstone, 1998; Hietanen, 1999, 2002; Langton & Bruce, 1999).

Both in the VPT and the gaze cueing paradigms, the other person is presented centrally on the screen and the typical effects on task performance indicate that participants extracted directional information from the social cue. The processing that follows the extraction of directional information is however usually interpreted slightly differently between these two paradigms. In the gaze cueing paradigm, the effect is traditionally explained in terms of a participant's attentional shift to where the other person is looking at (attentional cueing), while in the VPT paradigm the effect is discussed in terms of mentalizing about what the other person is seeing (for a discussion, see Heyes, 2014).The results from both strands of research have, however, been taken as evidence that we *automatically* compute where or what someone else is looking.

The notion of automaticity calls for a closer examination. Automaticity is a complex concept in cognitive sciences which can be defined based on three characteristics (Moors & De Houwer, 2006): (1) the process is unintentional (i.e., “uncontrolled in terms of the goal to engage in the process”, p. 309), (2) the process is efficient (or effortless; i.e., “consumes little or no processing resources or attentional capacity”, p. 317), and (3) the process is stimulus driven (i.e., “produced by the mere presence of the stimulus”, p. 308). In relation to the first characteristic, there is evidence that the computation of where/what someone else is looking at occurs even when it is not necessary to do so and even at the cost of task performance: in both the gaze cueing paradigm (Driver et al., 1999; Friesen & Kingstone, 1998; Mansfield, Farroni, & Johnson, 2003) and the VPT paradigm (Samson et al., 2010, Experiment 3; Santiesteban, Catmur, Coughlan Hopkins, Bird, & Heyes, 2013, Experiment 2), participants are not instructed to pay attention to the gazer and the gaze direction is not predictive of the location of the targets. Despite these factors, participants still compute where/what the gazer is looking at.

Secondly, it has been shown with both the gaze cueing and the VPT paradigms that the computation of where/what someone else is looking at is not suppressed or down-modulated when participants are in a dual-task situation, supporting the view that the computation is “effortless” (Hayward & Ristic, 2013; Law, Langton, & Logie, 2010; Qureshi, Apperly, & Samson, 2010).

However, regarding the third characteristic, i.e., the triggering conditions, the results so far are less clear and show apparently discrepant findings across the VPT and the gaze cueing paradigms. Indeed, one puzzling finding is that in the VPT paradigm the computation of what the other person sees is found when the gazing person is presented simultaneously with the object(s) to be processed, while in the gaze cueing paradigm the attentional cueing effect is only found when the gaze cue is presented between 50 and 800 ms prior to the object (i.e., with a Stimulus Onset Asynchrony [SOA] > 0; Frischen, Bayliss, & Tipper, 2007) but not when the gaze cue is presented simultaneously with the object (i.e., with a SOA = 0 ms ; Xu, Tanaka, & Mineault, 2012). In other words, in the gaze cueing paradigm (unlike in the VPT paradigm), the gazer has to be presented first alone without the competition of other objects for the cueing effect to be observed. Such discrepant findings could be interpreted in two ways. Firstly, the computation involved in the gaze cueing and the VPT paradigms could be qualitatively different (for example, the effect observed in the VPT paradigm could be linked to the drawing of a line of sight leading to a more robust or sustained effect while the effect observed in the gaze cueing paradigm could be due to a more simple attentional shift, non-mentalizing process; see Furnaletto, Becchio, Samson, & Apperly, in press; Heyes, 2014; Ramsey, Hansen, Apperly, & Samson, 2013; Samson et al., 2010; Schurz et al., 2015, for a discussion). Secondly, and irrespectively as to whether the same computation is at play in both paradigms, the attentional system could give a higher priority to the other person in the VPT paradigm than in the classic gaze cueing paradigm. A higher priority to the other person in the VPT paradigm would mean that the processing of the other person (more specifically the extraction of the directional cue and the subsequent processing of where or what the person is looking at) could withstand better the competition for attention from other objects in the scene. Importantly, this second explanation would indicate that the mere presence of another person is, in fact, not sufficient to compute what or where the other person is looking at, and that instead such a computation is dependent on contextual factors that allow for attention to give a priority to the other person. The current study focused on this latter hypothesis with the aim to understand the nature of the relevant contextual factors and whether these factors need necessarily to be social factors.

Concretely, we modified the classic gaze cueing paradigm by adding, in a stepwise fashion, features of the VPT paradigm without adding, however, the perspective-taking instructions. By doing so, we wanted to see whether any of these additional features would lead to the observation of a gaze cueing effect at an SOA of 0 ms or whether the attentional system prioritizes the gazer over other competing objects only in the social context of perspective taking.

A closer examination of the discrepant features across the VPT and classic gaze cueing paradigms (see Figure 1) highlighted three elements other than the perspective-taking context, that could explain why no gaze cueing effect is observed when a 0 ms SOA is used while an influence of the other person is observed in the VPT paradigm at such an SOA.

The first discrepant element is related to the nature of the stimuli used in the two paradigms. Visual salience (e.g., luminance contrast) is known to influence the bottom-up processes that capture our attention to certain features of the scene (Treisman & Gelade, 1980). The absolute visual salience of the gazer as well as its relative salience compared to other elements in the scene may thus have an impact on the extent to which attention is captured by the gazer and where/what he is looking at. The stimuli of the VPT paradigm are quite different to the ones that are classically used in the gaze cueing paradigm in that the avatar contrasts sharply in terms of luminance (i.e., dark hair and black shirt) with the rest of the scene (i.e., bright blue walls and bright red discs pinned on the wall). It is thus possible that the visual appearance of the stimuli of the VPT paradigm was more optimal to orient attention to the gazer and then to where/what he is looking. The visual salience of the directional information provided by the gazer is another factor that may trigger the computation of where/what another person is looking when an SOA of 0 ms is used. The stimuli of the VPT paradigm distinguish themselves from the ones usually used in the gaze cueing paradigm in that the directional information is conveyed by a full body avatar whose head and body are congruently oriented sideways (and with the eyes barely visible). In contrast, in the gaze cueing paradigm directional information is mainly provided by the eyes themselves (sometimes also by the head orientation, e.g., Langton & Bruce, 1999). Furthermore, the directional information provided by the gazer is sometimes found to be more salient when different directional cues (i.e., head and gaze) are oriented in the same direction than when they point to opposite directions (Langton, 2000; Langton & Bruce, 2000; but see Hietanen, 1999, 2002; Pomianowska, Germeys, Verfaillie, & Newell, 2012). The focus on body orientation and the congruency of the directional information across body, head and gaze cues in the VPT paradigm may thus facilitate the extraction of the directional information and hence facilitate the computation of where/what the other person is looking at.

The second element is the overall processing time required across the two types of paradigms. The overall reaction times for participants to judge their own perspective in the VPT paradigm are about 100 to 300 ms longer than the overall reaction times to detect or identify the target in the classic gaze cueing paradigm (Frischen et al., 2007). The longer processing time in the VPT paradigm may provide critical extra time for computing the gaze direction of the other person and prioritizing attention to where/what the other person is looking at.

Finally, the third element relates to differing task instructions across the two paradigms. Task instructions are known to influence how attention is deployed on the task stimuli (Posner, 1980). In the gaze cueing paradigm, participants are asked to detect or identify an object. Thus, the task instructions do not direct attention to the gazer but to the surrounding objects. In the VPT paradigm, participants are asked to judge what they see. Even though this instruction does not directly direct the participants’ attention to the gazer and should also direct attention to the surrounding objects, it has been shown that merely asking participants to focus on their own perspective directs their attention to the other person because they construe their perspective as distinct from the other person's perspective (Abbate, Isgrò, Wicklund, & Boca, 2006; Gendolla & Wicklund, 2009; Hass, 1984; Stephenson & Wicklund, 1983, 1984). Here the social mind-set induced by the self-perspective instruction may thus play a crucial role for attention prioritization. We examined whether such prioritization could be achieved by a non-social task instruction that directs attention to the gazer.

In a series of three experiments, we examined whether each of these elements plays a causal role in the generation of a gaze cueing effect at an SOA of 0 ms, indicating that a higher priority is given to the gazer and where/what he is looking at compared to any other stimulus presented simultaneously. Experiment 1 tested for the role of visual salience (luminance contrast and salience of directional information) by using the same stimuli as the ones used in the VPT paradigm by Samson et al. (2010) in a gaze cueing paradigm. Experiment 2 examined the role of processing time by equating the overall latencies of the gaze cueing paradigm with that of the VPT. Experiment 3 examined the effects of task instructions on attentional deployment by making it necessary to look at the gazer in the gaze cueing paradigm without perspective-taking instructions. In all three experiments, we contrasted participants’ performance (speed and accuracy) at identifying a target when the target appeared in a location congruent with the other person's gaze direction versus when the target appeared in a location incongruent with the person's gaze direction. The presence of a significant gaze congruency effect was taken as evidence that participants computed where/what the other person is looking at. All three experiments were approved by the ethics committee of the Psychological Sciences Research Institute of the Université catholique de Louvain.

## Experiment 1

Experiment 1 aimed to test the hypothesis that the stimuli used in the VPT paradigm (Samson et al., 2010) attract attention more to the gazer (and, as a consequence, facilitate the computation of where/what he is looking at) than the stimuli classically used in the gaze cueing paradigm (e.g., Driver et al., 1999; Friesen & Kingstone, 1998; Hietanen, 1999) in terms of luminance contrast and ease of extraction of the directional information. In order to test this hypothesis, we developed a gaze cueing task with the visual stimuli of the VPT task. This allowed us to determine whether the visual settings of the VPT task play a critical role in producing a measurable gaze congruency effect at the 0 ms SOA.

We also manipulated the onset of the target relative to the presentation of the gaze cue with, on half of the trials, the target objects appearing simultaneously with the gaze cue (SOA = 0 ms) and, on the other half of the trials, the target objects appearing 300 ms after the gaze cue (SOA = 300 ms). We expected to replicate in the 300 ms SOA condition the findings that participants’ performance to process the targets when they appear in a location congruent with the location the gazer is looking at would be superior to when the targets appear at the opposite location (e.g., Driver et al., 1999; Friesen, Moore, & Kingstone, 2005; Friesen & Kingstone, 1998). Furthermore, if the visual appearance of the stimuli of the VPT task is what explains the influence of the other person in the VPT paradigm at the 0 ms SOA, we should also find in our modified gaze cueing task a gaze congruency effect in the 0 ms SOA condition.

### Method

#### Participants

A total of 26 healthy individuals with normal or corrected-to-normal vision participated in the experiment in return of 5 Euros (21 females, mean age: 24.22, age range: 18–31).

#### Apparatus

Stimuli were presented on a 17-inch monitor (1024 × 68, 85 Hz, Dell M782p) with the E-prime software (Psychology Software Tools, Pittsburgh, PA, USA) running on a Dell Pentium 4 (2.8 GHz) computer. Participants sat at a distance of approximately 40 cm from the screen.

#### Stimuli and procedure

**Figure 2.**
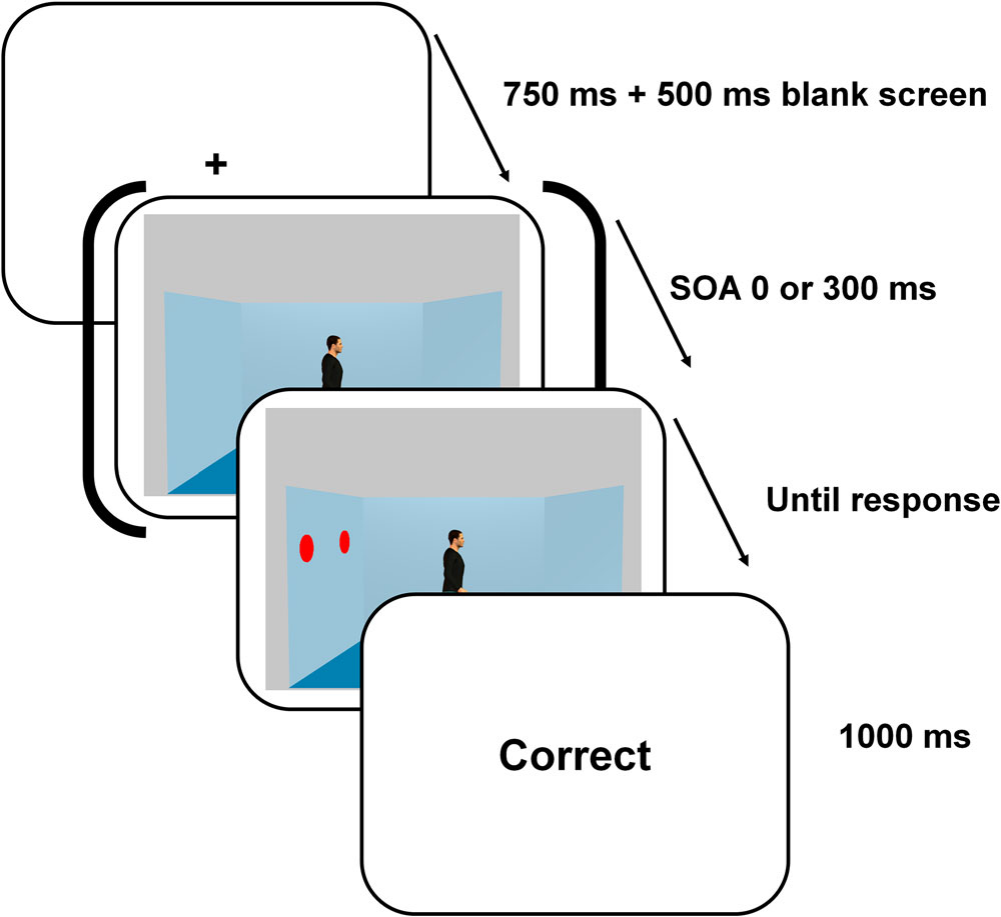
Illustration of the timing of the events on each trial of the modified gaze cueing task of Experiment 1. On this trial, participants had to press on the key “2” because two discs were visible in the room. The last screen displayed the feedback about participant's accuracy of response.

For each trial, participants saw first a fixation cross displayed for 750 ms followed by a 500 ms blank. They then saw a scene (19° × 11.5°) with an avatar (the gazer; 1.5° × 8°) positioned in the centre of a blue room. The avatar was always gender congruent with the participants’ gender. The avatar faced either the left or the right wall (see Figure 2). One or two red discs (the target object(s); 0.7° × 1.5° each, 1:1 probability) were presented on one of the lateral walls at 6.5° from the gazer. Participants were instructed to press “1” or “2” on the numerical keypad when, respectively, 1 or 2 red discs were pinned on the wall. Directly following the participant's response, a feedback “Correct”, “Incorrect”, or “No response” was presented for 1 s. A “No response” feedback was presented after 2 s had elapsed without a response from the participant.

On half of the trials, the discs were displayed simultaneously with the gazer and the background walls (0 ms SOA condition) whereas in the other half of the trials the red disc(s) appeared 300 ms after the presentation of the gazer and the background walls (300 ms SOA condition). Furthermore, on half of the trials, the discs were presented in the location the gazer was looking at (congruent gaze condition) whereas, on the other half of the trials, the discs appeared on the opposite wall (incongruent gaze condition). Forty-eight trials were presented in each of the four conditions that resulted from this 2 (SOA) × 2 (Congruency) design. The trials were presented in random order across two blocks of 96 trials preceded by a 32 trials practice block. The task lasted approximately 12 minutes.

### Result

Reaction times (RT) for correct responses and error rates (ER) were combined into an inverse efficiency score (IES), a unique measure of performance that merges ERs and RTs by weighting the average RT by the ER (IES = RT / (1-ER); Townsend & Ashby, 1978). Using the IES allows to homogenize the different patterns of speed/accuracy trade-offs within a group of individuals and to compare several groups via a unique measure. However, the IES presents the main disadvantage that the RTs are non-linearly multiplied (almost exponentially) as the ERs increase. This led Bruyer and Brysbaert (2011) to argue that the use of the IES should be avoided if there is more than 10% of erroneous responses (ER = .10). The observed percentages of errors indicated that it was statistically appropriate to use the IES in the present data analyses. Separate results for the RTs and ERs, and the full ANOVA on the IES, can be found in the Supplementary information S1. Table 1 shows the mean IES and RTs across experimental conditions.

**Table 1:** Mean and standard deviation of IES and RT for congruent and incongruent trials across experiments.

*M*(SD)	IES				RT			
SOA	0 ms		300 ms		0 ms		300 ms	
Congruency	Cong	Incong	Cong	Incong	Cong	Incong	Cong	Incong
Exp 1	506 (59)	505 (61)	428 (53)	446 (63)	500 (62)	498 (61)	420 (52)	440 (64)
Exp 2	622 (84)	627 (89)	552 (69)	583 (80)	615 (83)	614 (82)	549 (68)	575 (79)
Exp 3	811 (149)	864 (169)	607 (130)	645 (130)	760 (127)	797 (144)	566 (107)	588 (114)

Erroneous responses (1.4% of the data) and response omissions due to the timeout procedure (0.02% of the data) were eliminated from the data set when computing the median RTs. One participant's overall accuracy was 3 SD below the mean accuracy of the group and was thus removed from the analyses.

**Figure 3.**
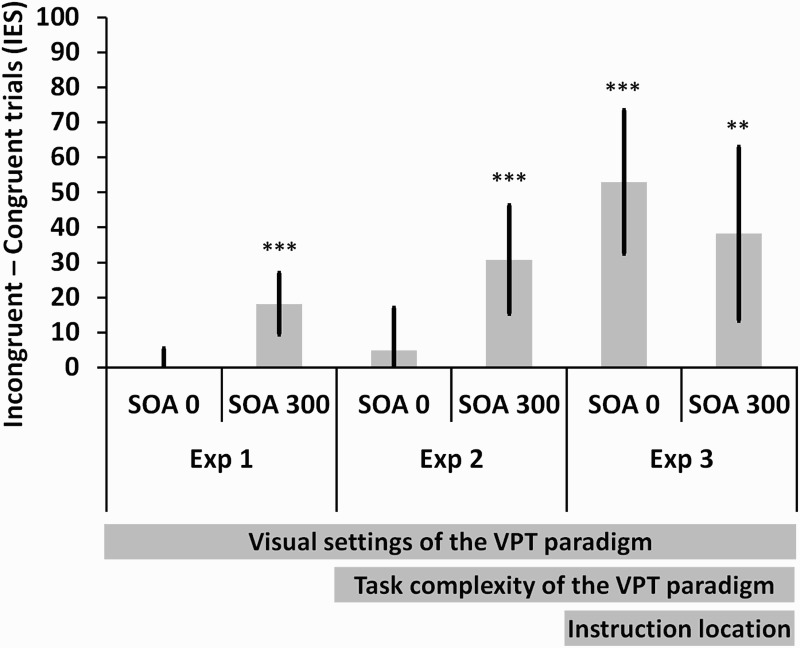
Gaze congruency indexes across Experiment 1, 2, and 3 in the modified gaze cueing paradigms. SOA = stimulus onset asynchrony, IES = inverse efficiency score. Error bars indicate the 95% confidence intervals. ** *p *< .01, *** *p *< .001.

As we were specifically interested in measuring the gaze congruency effect, we computed a *gaze congruency index* by subtracting the mean IES on congruent gaze trials from the mean IES on incongruent trials. The gaze congruency index was significantly different from 0 in the 300 ms SOA condition, *t*(24) = 4.077, *p *< .001, *d *= 0.82, but not in the 0 ms SOA condition, *t*(24) < 1, *p *= .655, *d *= 0.09. Furthermore, the gaze congruency index in the 300 ms SOA condition was significantly greater than the gaze congruency index in the 0 ms SOA condition, *t*(24) = 4.013, *p *= .001, *d *= 0.80 (see Figure 3).

These results replicate previous findings (e.g., Driver et al., 1999; Friesen et al., 2005; Friesen & Kingstone, 1998) indicating that participants’ performance was influenced by where/what a gazer is looking at and showed a gaze-cuing effect when using an SOA of 300 ms. Most importantly, despite the fact that we used the visual stimuli proper to the VPT task, we found no evidence of gaze cueing at an SOA of 0 ms. The nature of the stimuli used in the VPT task is thus not what triggers the computation of where/what the other person is looking at.

## Experiment 2

Experiment 2 aimed to test the hypothesis that the task complexity of the VPT task allows for extra time for the influence of the gazing avatar to start and modulate the task performance. In order to test this hypothesis, we developed a gaze cueing task that was matched in terms of overall response time to the VPT task. We also ensured that the stimuli used in the VPT and the gaze cueing tasks were matched in terms of visual appearance. Furthermore, as in Experiment 1, we manipulated the SOA (0 ms versus 300 ms). We expected to replicate the gaze congruency effect at an SOA of 300 ms but the critical question was whether this effect would now also be found at an SOA of 0 ms. If the increased task complexity of the novel gaze cueing task delays participants’ responses and through this gives a better opportunity for attention to be attracted to the gazer and where/what he is looking at, then we should find a significant gaze congruency effect with an SOA of 0 ms.

### Method

#### Participants

A total of 26 healthy individuals with normal or corrected-to-normal vision participated in the experiment in return of 8 Euros (17 females, mean age: 21.50, age range: 18–30). An additional 26 participants took part in the control VPT task (17 females, mean age: 21.57, age range: 18–28).

#### Apparatus

Identical to Experiment 1.

#### Stimuli and procedure

##### The gaze cueing task

Participants were presented with similar room displays as the ones used in Experiment 1. The room included one centrally positioned avatar facing either the left or the right wall and no discs^1^

**Figure 4.**
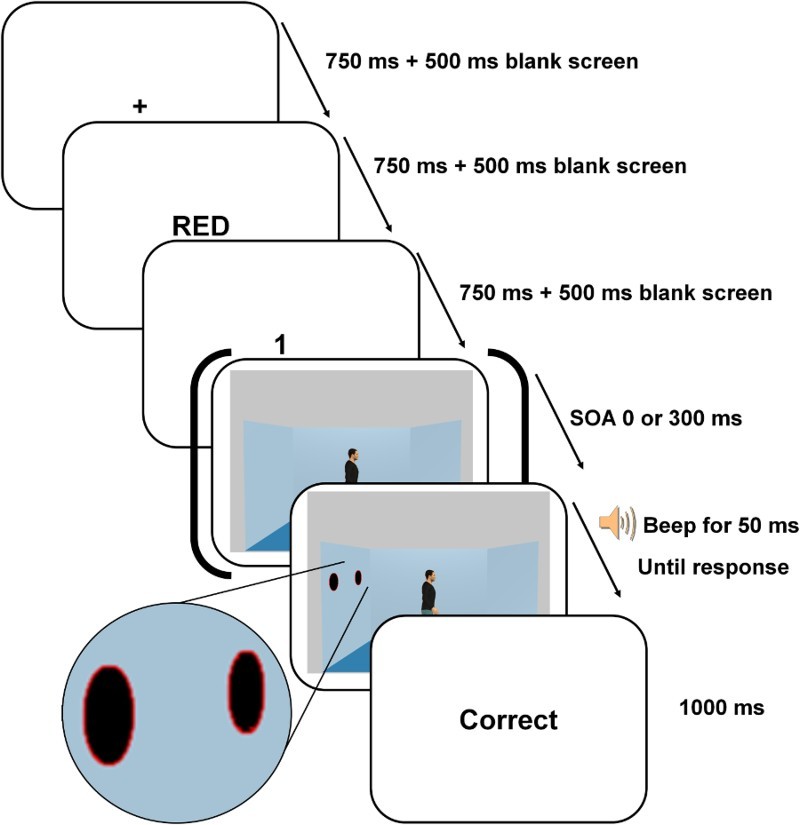
Illustration of the timing of the events on each trial of the modified gaze cueing task of Experiment 2. On this trial, participants had to judge whether there was one disc with a red border (see magnified view of the discs used) visible in the room. The last screen displayed the feedback about participant's accuracy of response.

^1^The trials without disc were used as catch trials. on the walls or one to two discs pinned on one of the walls. There were two main changes to the task (see Figure 4). (A series of pilot tests indicated that these were efficient ways to match the overall response times and task complexity across the gaze cueing and VPT tasks.) Firstly, the discs were not red anymore but were black with a fine red or green contour. Secondly, the participants were asked to verify whether two prompts presented before the room was displayed matched the content of the room. The sequence of events within a trial closely matched the sequence of events in the classic VPT task (Samson et al., 2010). More specifically, participants were first shown a colour prompt for 750 ms indicating which discs they had to take into account (“RED” meant that participants should only take into account the black discs with a red contour while “GREEN” meant that they had to take into account the black discs with a green contour). After a 500 ms blank screen, participants were presented with a number prompt (ranging from 0 to 2) for 750 ms. After a 500 ms blank screen, the room was displayed and the participants had to indicate whether the number prompt matched the number of discs with the prompted colour contour displayed in the room by pressing the upward arrow (yes) or downward arrow key (no). For example, following the prompts “RED” and “2”, participants had to say whether there were two black discs with a red contour in the room or not (“yes” or “no”). A 50 ms auditory signal was displayed informing when participants could respond. This feature was added to prevent participants from confusing trials from the 0 ms SOA condition with no disc in the room with trials from the 300 ms SOA condition (in which the appearance of the discs is delayed). Directly following the participant's response, a feedback “Correct”, “Incorrect”, or “No response” was presented for 1 s. A “No response” feedback was presented after 2 s had elapsed without a response from the participant.

On one half of the trials, the black disc(s) were displayed simultaneously with the gazer and the background walls (0 ms SOA condition) whereas on the other half of the trials the black disc(s) appeared 300 ms after the presentation of the scene (300 ms SOA condition). Furthermore, on half of the trials, the target discs appeared at the location gazed at (congruent gaze condition) whereas on the other half of the trials, the discs appeared on the opposite wall (incongruent gaze condition). There were 32 matching trials and 32 mismatching trials in each of the four experimental conditions = 2 (SOA) × 2 (Congruency). The two types of colour prompts (“RED” or “GREEN”) were equally distributed across all experimental conditions. As in the original VPT paradigm, mismatching trials were discarded from the analysis due to concerns that they may artificially inflate the gaze congruency effect (the number prompts in the mismatching trials of the congruent gaze condition do not correspond to either the number of red, green, or total number of discs and are thus particularly easy to reject). In addition, 64 filler trials with no disc or with discs on the two lateral walls were added to balance the occurrences of each number prompt (0, 1, or 2) across conditions; these filler trials were removed from the analyses. The trials were presented in random order across four blocks of 80 trials preceded by a 40 trials practice block. The task lasted approximately 32 minutes.

##### The control VPT task

A VPT task modified to closely match the visual and design settings of the gaze cueing task was added to directly compare overall reaction times and performance between the two paradigms. The VPT task was identical to the gaze cueing task except that the colour prompt was replaced by a perspective prompt indicating which perspective participants had to judge (“YOU” or “SHE”/“HE”). Participants had hence to judge whether the number prompt matched the number of discs that either they themselves (critical self-perspective trials) or the avatar (filler other-perspective trials)^2^

^2^Filler other-perspective trials are included to ensure that participants maintain a perspective-taking mind-set. If only self-perspective-taking trials are included, after a certain time participants risk to construe the task more like in the gaze cueing task (counting the discs) rather than as a perspective-taking task (what do “I” see). could see (see Figure 4). The colour of the disc contours varied as in the gaze cueing task but was not relevant for the VPT task. The gaze congruent condition corresponded to trials where participants had to judge their own perspective (“YOU” prompts) and where the discs appeared in the line of sight of the avatar. The gaze incongruent condition corresponded to trials where participants had to judge their own perspective (“YOU” prompts) and where the discs appeared on the opposite wall to the one the avatar was gazing at. The design (2 (SOA) × 2 (Congruency)) was identical to the gaze cueing task, including in terms of the number of matching, mismatching, and filler trials, which were identically distributed across the four experimental conditions. The two types of perspective prompts (“YOU” or “HE”/“SHE”) were equally distributed across all experimental conditions. The task lasted approximately 32 minutes.

### Result

In order to verify that the overall response times and overall performance was matched between the gaze cueing task and the VPT task, we conducted two *t*-tests for independent samples with the task condition as a between-subjects variable. The overall RT (averaged over Congruency and SOA) in the gaze cueing task (*M *= 601 ms) was not significantly different from the overall RT in the VPT task (*M *= 546 ms), *t*(48) = 1.635, *p *= .108. RTs on correct responses and ERs were then merged to use the IES. The overall performance, as measured with IES, in the gaze cueing task (*M *= 614) was not significantly different from the overall performance in the VPT task (*M *= 578), *t*(48) < 1, *p *= .334. Thus, the gaze cueing and VPT tasks were now matched in terms of processing time and efficiency.

Full results of the VPT task (for the RTs, ERs, and IES) are presented in the Supplementary information S2. We present below the results of the gaze cueing task.

Erroneous responses (1.4% of the data) and response omissions due to the timeout procedure (0.09% of the data) were eliminated from the data set when computing the median RTs. RTs on correct responses and ERs were merged to use the IES. Separate results for the RTs and ERs, and the full ANOVA on the IES, can be found in the Supplementary information S2. One participant's overall accuracy was 3 SD below the mean accuracy of the group and two participants’ congruency index were 3 SD higher than the mean congruency index of the group; these participants were thus removed from the analyses.

The gaze congruency index (computed in the same way as in the previous analyses) was significantly different from 0 in the 300 ms SOA condition, *t*(22) = 3.911, *p *= .001, *d *= 0.81, but not significantly different from 0 in the 0 ms SOA condition, *t*(22) < 1, *p *= .438, *d *= 0.16 (see Figure 3 and Table 1 for the mean IES and RTs per experimental condition). Furthermore, the gaze congruency index was significantly higher in the 300 ms SOA condition than in the 0 ms SOA condition, *t*(22) = 2.712, *p *= .013, *d *= 0.57. These results replicate the gaze congruency effect with an SOA of 300 ms (e.g., Driver et al., 1999; Friesen et al., 2005; Friesen & Kingstone, 1998) despite the increase in processing time. Importantly, however, there was no gaze congruency effect with an SOA of 0 ms. Consequently, the hypothesis according to which the task complexity or the working memory load of the VPT task allows more time for triggering the computation of where/what another person is looking at an SOA of 0 ms is not supported.

## Experiment 3

Experiment 3 aimed to test the hypothesis that the different task instructions across the classic gaze cueing paradigm and the VPT paradigm lead to a different attentional deployment on the scene. Through the perspective-taking instructions of the VPT task, attention would be deployed on the scene in such a way that it encompasses the gazer (and hence what he is looking at) even when participants judge their own perspective. Several studies suggest that this may result from the fact that perspective taking is about differentiating the self from another and that even when we think about the self, attention is naturally drawn to the other (e.g., Abbate et al., 2006; Gendolla & Wicklund, 2009; Hass, 1984; Stephenson & Wicklund, 1983). In contrast, the instructions of a classic gaze cueing task only draw attention to the objects present in a scene. In order to test this hypothesis, we modified the gaze cueing task so that attention is artificially drawn to the location of the avatar (importantly, however, without explicitly asking to pay attention to the avatar). This was achieved by superimposing the instruction prompts on the avatar. We again manipulated the SOA (0 versus 300 ms).

We expected to replicate the gaze congruency effect when the SOA is 300 ms and, as there are now two sources of attentional narrowing down onto the gazer (i.e., constricting attentional resources to a small portion of the visual field; Eriksen & Yeh, 1985), one source being the presentation of the gazer prior to the peripheral objects and the other source being the location of the task instructions, we could also expect that for these reasons the gaze congruency effect would be stronger than in Experiments 1 and 2. Furthermore, the crucial question was whether our task modifications were sufficient to find the gaze congruency effect at an SOA of 0 ms.

Note that we also ran again a control experiment in which we used the modified location of the task instruction in the classic VPT paradigm. The results showed that the influence of the other person in that paradigm could still be observed under those modified conditions (see Supplementary information S4).

### Method

#### Participants

A total of 25 healthy individuals with normal or corrected-to-normal vision participated in the experiment in return of 8 Euros (19 females, mean age: 21.70, age range: 18–28).

#### Apparatus

Identical to Experiment 1.

#### Stimuli and procedure

**Figure 5.**
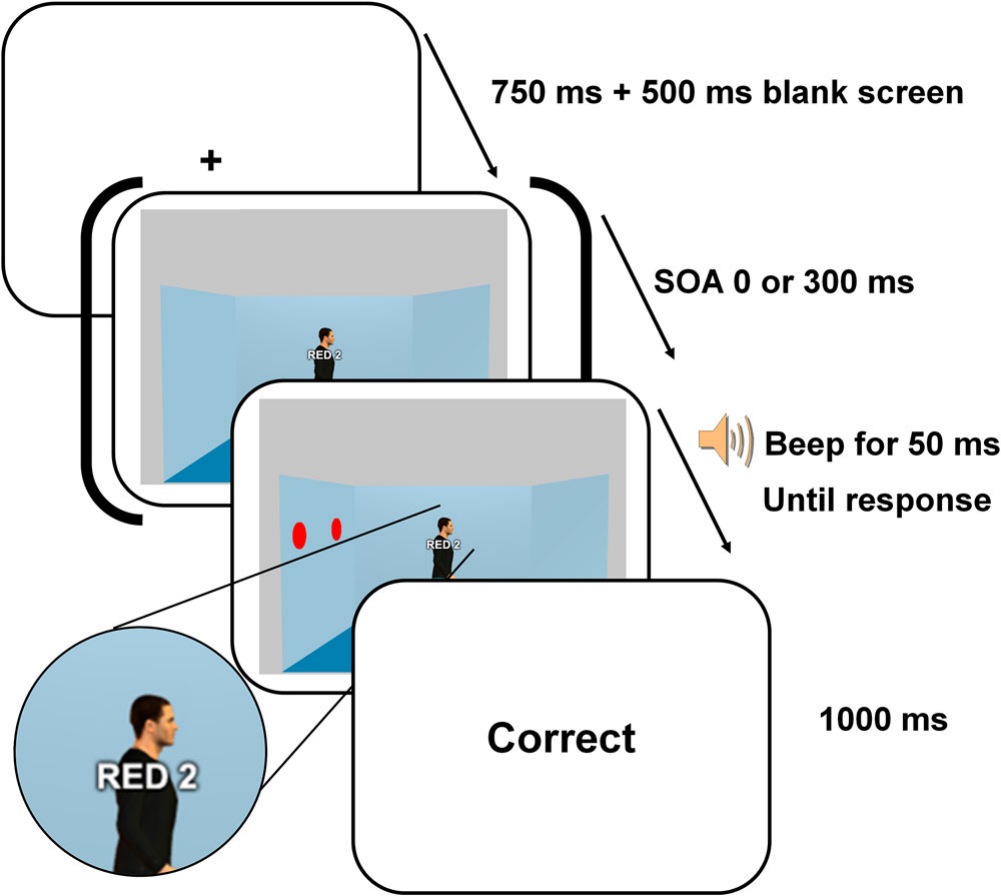
Illustration of the timing of the events on each trial of the modified gaze cueing task of Experiment 3. On this trial, participants had to judge whether there were two red discs visible in the room (see magnified view of the colour and number prompts superimposed on the avatar). The last screen displayed the feedback about participant's accuracy of response.

Participants were presented with the same stimuli and instructions as in Experiment 2 except for four changes made to the task. Firstly, the discs were not black with a red or green contour but were fully coloured in red or green (see Figure 5). Secondly, the colour and number prompts were not presented before the room was displayed but were presented simultaneously with the room, superimposed on the avatar's chest. This was done to force attention to be deployed on the avatar (without referring to perspective taking, however). Thirdly, the “GREEN” colour prompt was changed to “ALL” and meant that participants had to take into account all colours of discs. For example, participants were shown the room, read “ALL 2” on the avatar's chest, and thus had to say whether there were two discs in the room or not, no matter their colour. This change was made to match more closely the effects of the perspective prompts on attention deployment (“ALL” matching the “SELF” prompt by encompassing all discs and “RED” matching the “SHE”/“HE” prompt by encompassing sometimes only a subset of discs). Fourthly, the discs displayed on the same wall were either all red or all green to reduce the cognitive demand of selective attention in the “RED” colour instruction condition. (Otherwise, the green disc would have been a nearby, and thus potent, distractor.)

The design (2 (SOA: 0 vs. 300 ms) × 2 (Congruency: congruent vs. incongruent gaze)) was identical to Experiment 2, including the number of matching, mismatching, and filler trials, which were identically distributed across the four experimental conditions. The two types of colour prompts (“RED” or “ALL”) were equally distributed across all experimental conditions. The task lasted approximately 22 minutes and contained four blocks of 80 trials preceded by a practice block of 24 trials.

### Result

RTs on correct responses and ERs were merged to use the IES. Separate results for the RTs and ERs, and the full ANOVA on the IES, can be found in the Supplementary information S3.

Erroneous responses (2.1%) and response omissions due to the timeout procedure (0.02% of the data) were eliminated from the data set when computing the median RTs. Matching trials with red colour instructions were presented with a zero digit prompt (“RED 0”) when the discs were all green (unlike in Experiment 2, discs were either all green or all red). Several participants reported that the absence of red discs following “RED 0” led to a prepotent “no” answer, which actually had to be suppressed to accurately respond “yes”. This artificially hampered task performance (the mean accuracy on these trials was 3 SD below the overall accuracy) and therefore those trials were removed from the analyses.

The gaze congruency index (computed in the same way as in the previous analyses) was significantly different from 0 in the 300 ms SOA condition, *t*(24) = 3.025, *p *= .006, *d *= 0.60, and, this time, also in the 0 ms SOA condition, *t*(24) = 5.089, *p *< .001, *d *= 1.01 (see Figure 3 and Table 1 for the mean IES and RTs per experimental condition). Moreover, the gaze congruency index in the 300 ms SOA condition was not significantly different from the gaze congruency index in the 0 ms SOA condition, *t*(24) < 1, *p *= .458, *d *= 0.15. The comparison of the gaze congruency indexes across Experiment 1 in the 300 ms SOA condition (which is representative of the average 16 ms gaze cueing effect calculated in the review by Lachat, Conty, Hugueville, & George, 2012) and Experiment 3, showed that the index was significantly higher in Experiment 3, *t*(49) = 3.115, *p *= .003, *d *= 0.89.

Thus, these results replicate the finding of the gaze congruency effect with an SOA of 300 ms (e.g., Driver et al., 1999; Friesen et al., 2005; Friesen & Kingstone, 1998) and show that this effect was even further enhanced. Importantly, the gaze congruency effect was found for the first time with an SOA of 0 ms. This finding strongly indicates that drawing attention to the location of the gazer through task instructions plays a causal role in the computation of where/what another person is looking.

## General Discussion

Across three experiments in which participants completed a gaze cueing task, our results replicated the well-known finding that participants perform better in identifying a target presented in the line of sight of a gazer than a target presented outside of its line of sight when the gazer is presented 300 ms prior to the target (i.e., with an SOA of 300 ms; Driver et al., 1999; Friesen & Kingstone, 1998). Furthermore, we showed that the presence of a gaze congruency effect at an SOA of 0 ms cannot be systematically found despite matching the gaze cueing task in terms of the appearance of the stimuli (Experiment 1) or processing time (Experiment 2) with the VPT task. The gaze congruency effect at an SOA of 0 ms was only found when the instructions specifically directed participants’ attention to the avatar's location by having a part of the instructions superimposed on the avatar (Experiment 3). Altogether, this pattern of results provides important insights into the conditions in which we process where/what another person is looking at.

### Revisiting the automaticity claim

Our results show that once attention is drawn to a person (even when there is no explicit instruction to take into account that person's visual experience), what is in the line of sight of the person is automatically computed and influences judgments we make about what we ourselves see. This is truly independent of whether participants are placed in a gaze cueing or a VPT task. Importantly, however, our results show that whether attention is drawn to the person in the first place is context-dependent: the social mind-set created by a perspective-taking task (even when participants are only required to judge their own perspective) seems to naturally draw attention to the other person; whereas in a gaze cueing task, attention has to be drawn by external factors such as the prior presentation of the gazer alone without competing stimuli (as with the classic 300 ms SOA) or the presentation of task-relevant information in the same location as the avatar (as in Experiment 3^3^

^3^It should be noted that while we can conclude that the presentation of task-relevant information in the same location as the avatar is critical to produce the gaze congruency effect at the 0 ms SOA, we cannot exclude the possibility that the effect resulted, in fact, from a combined effect of the task instruction with both/either the visual settings and/or the task complexity.). This means that it is not the mere presence of another person in our field of view that triggers attentional cueing or line of sight computation but the act of looking at or attending to another person. This has implications for the question of the level of automaticity with which we compute where/what someone else is looking at.

In relation to the three characteristics of an automatic process defined by Moors and De Houwer (2006) (i.e., whether the process is unintentional, efficient, and stimulus driven), previous studies have shown evidence that, both for the gaze cueing and the VPT paradigms, the computation of where/what someone else is looking at occurs even when it is not required for the efficient task performance (Driver et al., 1999; Friesen & Kingstone, 1998; Mansfield et al., 2003; Samson et al., 2010; Santiesteban et al., 2013) and even when participants are in a dual-task situation (Hayward & Ristic, 2013; Law et al., 2010; Qureshi et al., 2010).

However, regarding the third characteristic of automaticity, our results here clearly show that the mere presence of the other person is not sufficient to trigger the computation of where/what that person is looking at. Hence, computing where/what another person is looking at is not stimulus driven but context-dependent. Thus, in a situation where a person is surrounded by objects (like a classic gaze cueing task at an SOA of 0 ms), attention is probably distributed across the scene and this does not seem to be sufficient to trigger the computation of where/what another person is looking at. Instead, attention needs to prioritize the gazing person (compared to other objects in the scene) before the computation of what the gazing person is looking at can happen. The prioritization can be achieved in at least three contexts. First, in the context of the VPT task, attention seems to be narrowed down to the gazing person because of its high relevance for the perspective-taking instructions. The high relevance seems to suffice to prioritize the processing of the gazing person over the processing of the competing peripheral target and would explain why there is an effect of what the other person is seeing at an SOA of 0 ms with the VPT task. Second, in the context of the classic gaze cueing paradigm with an SOA of at least 50 ms, attention is narrowed down to the gazing person because it appears first alone without competing objects in the scene, which is why a gaze congruency effect can be consistently found at an SOA of 300 ms across all experiments. Third, in the context of goal-relevant information placed on the same location as the gazing person, attention is narrowed down to the area of the gazing person in order to read the instructions. This also seems to be sufficient to prioritize the processing of the gazing person over the competing peripheral target and explains why we found a gaze congruency effect at an SOA of 0 ms in Experiment 3 even though there was no social task instructions. In sum, the computation of where/what another person is looking at does not seem to occur when the gazing person and the peripheral target appear simultaneously unless attention is narrowed down to the gazing person through the influence of social or non-social factors.

Interestingly, the mere fact of judging one's own visual perspective is sufficient to trigger the computation of what another person is seeing. Following the seminal finding of Hass (1984), Wicklund and colleagues (Abbate et al., 2006; Gendolla & Wicklund, 2009; Stephenson & Wicklund, 1983, 1984) have repeatedly shown that, when we are asked to focus on ourselves, we are more prompt to take another person's perspective. These authors stated that such self-focus exacerbates our awareness of how distinct we are from others and how we are perceived by others, which increases the salience of the other person's perspective (Stephenson & Wicklund, 1983). This latter view is line with the observation that young children who fail to take another person's visual perspective are those who fail to differentiate the self from others (Flavell, Botkin, Fry, Wright, & Jarvis, 1968, 1981). Altogether, it seems likely that, in the VPT paradigm, when participants are explicitly asked to take their own visual perspective but not the avatar's visual perspective, they are put in a social mind-set that increases the salience of the gazer and thus the amount of attention deployed on it.

### Computing where someone is looking at or what someone is seeing?

Do participants compute a “mental” state (what the gazer “sees”) or do they simply extract a directional feature (where the gazer is looking at) that orients attention? Furthermore, do they compute the same in both types of paradigms? Some evidence suggests that participants compute a “seeing” mental state when observing the gazer in the gaze cueing paradigm (Teufel, Alexis, Clayton, & Davis, 2010) or in the VPT paradigm (Furnaletto et al., in press). Teufel et al. (2010) have shown, for example, that when the gazer wore goggles that were told to be opaque, participants’ processing of a subsequent target was much less affected by the gazer's head orientation than when the gazer wore similar goggles that were told to be transparent. Furnaletto et al. (in press) showed the same effect in the VPT paradigm: when making self-perspective judgements, participants were not influenced anymore by what was in front of the gazer if they knew that the gazer wore opaque goggles. Similarly, the gaze congruency effect in a gaze cueing paradigm is larger when participants attribute a higher degree of agency to the gazer (Wiese, Wykowska, Zwickel, & Müller, 2012). These results indicate that participants extract more than just a directional cue when observing the gazer in both the gaze cueing and the VPT paradigms. Other research shows, however, a more nuanced pictured with the report of no modulation of the gaze congruency effect when an obstacle (a barrier instead of opaque goggles) is present in the gazer's line of sight (Cole, Smith, & Atkinson, 2015). Further research is needed to investigate the possible interplays between extracting a directional cue and computing what the gazer is seeing as this may occur through separate processing stages (e.g., Ramsey et al., 2013; Wiese et al., 2012).

## Conclusion

Humans are sensitive to where and what other people are looking at. However, the mere presence of another person in the field of view is not sufficient to compute where/what the other person is looking at. In that sense, such a computation is not entirely stimulus-driven and hence not fully automatic. Instead, attention first needs to be narrowed down to the other person in order to prevent competition from other potential sources of attention capture or orienting. The social mind-set induced by perspective-taking instructions (even when one only focuses on his/her own perspective) provides a powerful trigger to compute where and what the other person is looking at by prioritizing attention to the other person. We showed also that non-social factors can help as well prioritizing attention to the other person and trigger the computation of where and what the other person is looking at. Such findings open new avenues for investigating the origin of spontaneous perspective-taking difficulties in some clinical populations (e.g., autism spectrum disorders; Senju, Southgate, White, & Frith, 2009; Senju et al., 2010) and ways this could be compensated for.
