# Supplementary material for: From gaze cueing to perspective taking: Revisiting the claim that we automatically compute where or what other people are looking at
Source: Vis cogn. 2016 Jan 24;23(8):1020–42. doi: 10.1080/13506285.2015.1132804 (PMC4743615; doi:10.1080/13506285.2015.1132804)
Supplement: Supplementary Information.docx [file pvis_a_1132804_sm0805.docx]

**Supplementary information S1: Additional results of the gaze cueing task in Experiment 1 (RT, ER, and IES)**

The same participant as in the analyses on the IES congruency index was removed (i.e., one participant whose overall accuracy was 3 *SD* below the mean accuracy of the group).

### Reaction times. Erroneous responses and response omissions due to the timeout procedure were eliminated from the data set when computing the median RTs.

A 2 (Congruency) x 2 (SOA) ANOVA was conducted on median RTs. The ANOVA revealed a significant main effect of Congruency, *F* (1, 24) = 13.838, *MSE* = 2,116, *p* = .001, η_p_² = .366, with slower RTs in incongruent trials than in congruent trials, a significant main effect of SOA, *F* (1, 24) = 480.578, *MSE* = 118,198, *p* < .001, η_p_² = .952, with slower RTs in the 0-ms SOA condition than in the 300-ms condition, and a significant Congruency x SOA interaction effect, *F* (1, 24) = 22.564, *MSE* = 3,080, *p* < .001, η_p_² = .485.

Similarly to the analyses conducted on IESs, we computed a gaze congruency index by subtracting the mean RT on congruent trials from that on incongruent trials. The gaze congruency index was not different from 0 in the 0-ms condition, *t* (24) < 1, *p* = .519, but significantly different from 0 in the 300-ms SOA condition, *t* (24) = 5.288, *p* < .001.

**Error rates.** A 2 (Congruency) x 2 (SOA) ANOVA was conducted on error rates. The ANOVA revealed no significant effect (all *F*s < 1, all *p*s > .425).

**Inverse efficiency scores.** A 2 (Congruency) x 2 (SOA) ANOVA was conducted on the IES. The ANOVA revealed a significant main effect of Congruency, *F* (1, 24) = 6.591, *MSE* = 1,715, *p* = .017, η_p_² = .215, with worse performance on incongruent trials than congruent trials, a significant main effect of SOA, *F* (1, 24) = 379.546, *MSE* = 118,656, *p* < .001, η_p_² = .941, with worse performance in the 0-ms SOA condition than in the 300-ms SOA condition, and a significant Congruency x SOA interaction effect, *F* (1, 24) = 16.102, *MSE* = 2,461, *p* < .001, η_p_² = .402.

**Supplementary information S2: Additional results from Experiment 2**

**Results of the gaze cueing task in Experiment 2**

The same participants as in the analyses on the IES congruency index were removed (i.e., one participant whose overall accuracy was 3 *SD* below the mean accuracy of the group and two participants whose congruency index were 3 *SD* higher than the mean congruency index of the group).

**Reaction times.** Erroneous responses and response omissions due to the timeout procedure were eliminated from the data set when computing the median RTs.

A 2 (Congruency) x 2 (SOA) ANOVA was conducted on median RTs. The ANOVA revealed a significant main effect of Congruency, *F* (1, 22) = 5.621, *MSE* = 3,537, *p* = .027, η_p_² = .204, with slower RTs in incongruent trials than in congruent trials, a significant main effect of SOA, *F* (1, 22) = 76.351, *MSE* = 61,906, *p* < .001, η_p_² = .776, with slower RTs in the 0-ms SOA condition than in the 300-ms condition, and a significant Congruency x SOA interaction effect, *F* (1, 22) = 10.404, *MSE* = 4,375, *p =* .004, η_p_² = .321.

Similarly to the analyses conducted on IESs, we computed a gaze congruency index by subtracting the mean RT on congruent trials from that on incongruent trials. The gaze congruency index was not different from 0 in the 0-ms condition, *t* (22) < 1, *p* = .833, but significantly different from 0 in the 300-ms SOA condition, *t* (22) = 3.751, *p* = .001.

**Error rates.** A 2 (Congruency) x 2 (SOA) ANOVA was conducted on ERs. The ANOVA revealed no significant effect (Congruency: *F* (1, 22) = 2.773, *MSE* = .001, *p* = .110, η_p_² = .112; SOA: *F* (1, 22) = 1.963, *MSE* = .001, *p* = .0175, η_p_² = .175; Congruency x SOA: *F* (1, 22) = 0.027, *MSE* < 0.001, *p* = .870, η_p_² = .001).

**Inverse efficiency scores.** A 2 (Congruency) x 2 (SOA) ANOVA was conducted on the IES. The ANOVA revealed a significant main effect of Congruency, *F* (1, 22) = 11.630, *MSE* = 7,336, *p* = .003, η_p_² = .346, with worse performance on incongruent trials than congruent trials, a significant main effect of SOA, *F* (1, 22) = 65.693, *MSE* = 73,717, *p* < .001, η_p_² = .749, with worse performance in the 0-ms SOA condition than in the 300-ms SOA condition, and a significant Congruency x SOA interaction effect, *F* (1, 22) = 7.355, *MSE* = 3,866, *p* = .013, η_p_² = .251.

**Results of the control VPT task in Experiment 2**

**Self-perspective trials only.**

As a reminder, only self-perspective trials were taken into account as these are the trials where computing what the avatar sees is task-irrelevant.

Erroneous responses (5.4% of the data) and response omissions due to the timeout procedure (0.24% of the data) were eliminated from the data set when computing the median RTs.

***Reaction times.*** The same participant as in the analyses on the IES congruency index was removed (i.e., one participant whose overall accuracy was 3 *SD* below the mean accuracy of the group). A 2 (Congruency) x 2 (SOA) ANOVA was conducted on median RTs. The ANOVA revealed a significant main effect of Congruency, *F* (1, 24) = 15.995, *MSE* = 39,204, *p* = .001, η_p_² = .204, with slower RTs in incongruent trials than in congruent trials, a significant main effect of SOA, *F* (1, 24) = 41.204, *MSE* = 207,025, *p* < .001, η_p_² = .632, with slower RTs in the 0-ms SOA condition than in the 300-ms condition, and a non-significant Congruency x SOA interaction effect, *F* (1, 24) = 1.222, *MSE* = 3,147, *p =* .280, η_p_² = .048.

Similarly to the analyses conducted on IESs, we computed a gaze congruency index by subtracting the mean RT on congruent trials from that on incongruent trials. The gaze congruency index was significantly different from 0 both in the 0-ms and the 300-ms SOA conditions, *t* (24) = 3.284, *p* = .003, *t* (24) = 2.226, *p* = .036, respectively.

***Error rates.*** The same participant as in the analyses on the IES congruency index was removed (i.e., one participant whose overall accuracy was 3 *SD* below the mean accuracy of the group). A 2 (Congruency) x 2 (SOA) ANOVA was conducted on ERs. The ANOVA revealed a significant main effect of Congruency, *F* (1, 22) = 6.894, *MSE* = .010, *p* = .015, η_p_² = .239, with more errors in incongruent trials than in congruent trials, no significant main effect of SOA, *F* (1, 22) < 1, *MSE* = .001, *p* = .580, η_p_² = .014, and no significant Congruency x SOA interaction effect, *F* (1, 22) < 1, *MSE* = .001, *p =* .874, η_p_² = .001.

Similarly to the analyses conducted on IESs, we computed a gaze congruency index by subtracting the mean ER on congruent trials from that on incongruent trials. The gaze congruency index was close to be significantly different from 0 both in the 0-ms and the 300-ms SOA conditions, *t* (22) = 1.908, *p* = .069, *t* (22) = 1.699, *p* = .103, respectively.

***Inverse efficiency scores.*** One participant’s overall accuracy was 3 *SD* below the mean accuracy of the group and was thus removed from the analyses. A 2 (Congruency) x 2 (SOA) ANOVA was conducted on the IES. The ANOVA revealed a significant main effect of Congruency, *F* (1, 24) = 21.526, *MSE* = 119,668, *p* < .001, η_p_² = .473, with worse performance on incongruent trials than congruent trials, a significant main effect of SOA, *F* (1, 24) = 20.322, *MSE* = 206,829, *p* < .001, η_p_² = .459, with worse performance in the 0-ms SOA condition than in the 300-ms SOA condition, and a non-significant Congruency x SOA interaction effect, *F* (1, 24) < 1, *MSE* = 2,045, *p* = .352, η_p_² = .036.

Similarly to the analyses of Experiment 1, we computed a gaze congruency index by subtracting the mean IES on congruent gaze trials from that on incongruent trials. The gaze congruency index was significantly different from 0 both in the 0-ms and 300-ms SOA conditions, *t* (24) = 3.632, *p* = .001, *d* = 0.73, *t* (24) = 2.878, *p* = .008, *d* = 0.58, respectively. Furthermore, the gaze congruency index in the 300-ms SOA condition was not significantly different from the gaze congruency index in the 0-ms SOA condition, *t* (24) < 1, *p* = .352, *d* = 0.19. These results replicate the attentional orienting effect at an SOA of 0 ms and, in addition, show for the first time that this effect is maintained when the SOA is increased to 300 ms.

**Analyses including the other-perspective trials**

One participant’s overall accuracy was 3 *SD* below the mean accuracy of the group and was thus removed from the analyses. The ANOVA 2 (Perspective instruction: self vs. other-perspective) x 2 (Congruency) x 2 (SOA) conducted on the IESs revealed a significant main effect of Congruency, *F* (1, 24) = 35.118, *MS*E = 1,336,430, *p* < .001, η_p_² = .594, with better performances on congruent perspectives trials than on incongruent perspectives trials, a main effect of Perspective, *F* (1, 24) = 14.695, *MSE* = 231,268, *p* < .001, η_p_² = .380, with participants being better at judging their own perspective than the avatar’s perspective, a significant Congruency x Perspective interaction effect, *F* (1, 24) = 21.210, *MSE* = 444,648, *p* < .001, η_p_² = .469, and a significant main effect of SOA, *F* (1, 24) = 35.982, *MS*E = 401,339, *p* < .001, η_p_² = .600, with worse performances in the 0-ms SOA condition than in the 300-ms SOA condition.

To explore the interaction between Congruency and Perspective, a series of paired t-tests tested the effect of Congruency on self- and other-perspective trials separately and the effect of Perspective on congruent and incongruent perspectives trials separately. Paired t-tests showed a significant Congruency effect on both self- and other-perspective trials, *t* (24) = 4.640, *p* < .001, *t* (24) = 5.575, *p* < .001, respectively, with better performances on congruent perspectives trials. There was a significant Perspective effect on congruent perspective trials, *t* (24) = 2.542, *p* = .018, with participants being better at judging the avatar’s perspective than their own perspective, and a significant the effect of Perspective on incongruent perspective trials, *t* (24) = 4.400, *p* < .001, with participants being better at judging from their own perspective and the avatar’s perspective. These results are in line with the results reported in the original paradigm (Samson et al., 2010).

**Supplementary information S3: Additional results of the gaze cueing task in Experiment 3**

**Reaction times**. Erroneous responses and response omissions due to the timeout procedure were eliminated from the data set when computing the median RTs.

A 2 (Congruency) x 2 (SOA) ANOVA was conducted on median RTs. The ANOVA revealed a significant main effect of Congruency, *F* (1, 24) = 23.471, *MSE* = 21,550, *p* < .001, η_p_² = .494, with slower RTs in incongruent trials than in congruent trials, a significant main effect of SOA, *F* (1, 24) = 519.219, *MSE* = 1,012,639, *p* < .001, η_p_² = .956, with slower RTs in the 0-ms SOA condition than in the 300-ms condition, and no significant Congruency x SOA interaction effect, *F* (1, 24) = 1.037, *MSE* = 1,697, *p =* .319, η_p_² = .041.

Similarly to the analyses conducted on IESs, we computed a gaze congruency index by subtracting the mean RT on congruent trials from that on incongruent trials. The gaze congruency index was significantly different from 0 both in the 0-ms and the 300-ms SOA conditions, *t* (24) = 3.704, *p* = .001, *t* (24) = 2.097, *p* = .047, respectively.

**Error rates.** A 2 (Congruency) x 2 (SOA) ANOVA was conducted on ERs. The ANOVA revealed a significant main effect of Congruency, *F* (1, 24) = 11.340, *MSE* = .004, *p* = .003, η_p_² = .321, with more errors in incongruent trials than in congruent trials, no significant main effect of SOA, *F* (1, 24) < 1, *MSE* = .001, *p* = .579, η_p_² = .013, and no significant Congruency x SOA interaction effect, *F* (1, 24) < 1, *MSE* = .001, *p =* .558, η_p_² = .014.

Similarly to the analyses conducted on IESs, we computed a gaze congruency index by subtracting the mean ER on congruent trials from that on incongruent trials. The gaze congruency index was close to be significantly different from 0 both in the 0-ms and the 300-ms SOA conditions, *t* (24) = 1.769, *p* = .090, *t* (24) = 2.009, *p* = .056, respectively.

**Inverse efficiency scores.** A 2 (Congruency) x 2 (SOA) ANOVA was conducted on the IES. The ANOVA revealed a significant main effect of Congruency, *F* (1, 24) = 55.994, *MSE* = 52,062, *p* < .001, η_p_² = .700, with worse performance on incongruent trials than congruent trials, a significant main effect of SOA, *F* (1, 24) = 462.004, *MSE* = 1,118,193, *p* < .001, η_p_² = .951, with worse performances in the 0-ms SOA condition than in the 300-ms SOA condition, and no significant Congruency x SOA interaction effect, *F* (1, 24) < 1, *MSE* = 1,377, *p* = .458, η_p_² = .023.

**Supplementary information S4: Additional experiment conducted with a control VPT task matched to the gaze cueing task of Experiment 3**

**Method**

**Participants**

25 healthy individuals with normal or corrected-to-normal vision participated in the experiment in return of 8 euros (19 females, mean age: 22.10, age range: 18-30).

### Apparatus

### Identical to Experiment 1.

**Stimuli and procedure**

Participants were presented with same stimuli as the gaze cueing task of Experiment 3 but with the prompts of the VPT task of Experiment 2. For example, participants were shown the room, read “YOU 2” on the avatar’s chest, and thus had to say whether they could see from their own perspective two discs in the room or not.

The design (2 (SOA: 0 vs. 300 ms) x 2 (Congruency: congruent vs. incongruent gaze)) was identical to Experiment 2, including in terms of the number of matching, mismatching, and filler trials, which were identically distributed across the 4 experimental conditions. The two types of perspective prompts (“YOU” or “SHE”/“HE”) were equally distributed across all experimental conditions. The task lasted approximately 22 minutes and contained 4 blocks of 80 trials preceded by a practice block of 24 trials.

**Results**

Erroneous responses (5.3% of the data) and response omissions due to the timeout procedure (0.03% of the data) were eliminated from the data set when computing the median RTs. RTs on correct responses and ERs were merged to use the IES. The results are presented in two sections: The first section presents the results where analyses were carried only on self-perspective trials to match the results – the congruency indexes in particular – with those obtained in the gaze cueing paradigm in Experiment 3. The second section present the results where analyses were carried on both the self- and other-perspective trials to match the results obtained in the original level-1 visual perspective-taking task (Samson et al., 2000).

**Analyses on the self-trials only**

**Reaction times**. Erroneous responses and response omissions due to the timeout procedure were eliminated from the data set when computing the median RTs. As a reminder, only self-perspective trials were taken into account as these are the trials where computing what the avatar sees is task-irrelevant.

A 2 (Congruency) x 2 (SOA) ANOVA was conducted on median RTs. The ANOVA revealed a significant main effect of Congruency, *F* (1, 24) = 54.716, *MSE* = 309,136, *p* < .001, η_p_² = .695, with slower RTs in incongruent trials than in congruent trials, a significant main effect of SOA, *F* (1, 24) = 205.559, *MSE* = 1,255,296, *p* < .001, η_p_² = .895, with slower RTs in the 0-ms SOA condition than in the 300-ms condition, and a significant Congruency x SOA interaction effect, *F* (1, 24) = 8.603, *MSE* = 23,286, *p =* .007, η_p_² = .264.

Similarly to the analyses of Experiment 1, 2 and 3, we computed a gaze congruency index by subtracting the mean RT on congruent trials from that on incongruent trials. The gaze congruency index was significantly different from 0 both in the 0-ms and the 300-ms SOA conditions, *t* (24) = 7.949, *p <* .001, *t* (24) = 4.308, *p* < .001, respectively.

**Error rates analyses.** A 2 (Congruency) x 2 (SOA) ANOVA was conducted on ERs. The ANOVA revealed a significant main effect of Congruency, *F* (1, 24) = 7.953, *MSE* = .031, *p* = .009, η_p_² = .370, with lower performances in incongruent trials than in congruent trials, no significant main effect of SOA, *F* (1, 24) < 1, *MSE* = .002, *p* = .420, η_p_² = .027, and no significant Congruency x SOA interaction effect, *F* (1, 24) < 1, *MSE* = .001, *p =* .597, η_p_² = .012.

Similarly to the analyses of Experiment 1, 2 and 3, we computed a gaze congruency index by subtracting the mean ER on congruent trials from that on incongruent trials. The gaze congruency index was significantly different from 0 both in the 0-ms and the 300-ms SOA conditions, *t* (24) = 2.295, *p* = .031, *t* (24) = 2.268, *p* = .033, respectively.

**Inverse efficiency scores.** A 2 (Congruency) x 2 (SOA) ANOVA was conducted on the IES. The ANOVA revealed a significant main effect of Congruency, *F* (1, 24) = 49.413, *MSE* = 692,650, *p* < .001, η_p_² = .673, with worse performance on incongruent trials than congruent trials, a significant main effect of SOA, *F* (1, 24) = 61.923, *MSE* = 1,465,766, *p* < .001, η_p_² = .721, with worse performances in the 0-ms SOA condition than in the 300-ms SOA condition, and a significant Congruency x SOA interaction effect, *F* (1, 24) = 10.735, *MSE* = 67,453, *p* = .003, η_p_² = .309.

Similarly to the analyses of Experiment 1, 2 and 3, we computed a gaze congruency index by subtracting the mean IES on congruent gaze trials from that on incongruent trials (for self-perspective trials only). The gaze congruency index was significantly different from 0 both in the 0-ms and 300-ms SOA conditions, *t* (24) = 4.490, *p* < .001, *d* = 0.90, *t* (24) = 6.999, *p* < .001, *d* = 1.40, respectively (see Figure 4(b)). However, the gaze congruency index in the 300-ms SOA condition was significantly smaller than the gaze congruency index in the 0-ms SOA condition, *t* (24) = 3.276, *p* = .003, *d* = 0.66. These results replicated the findings of attentional orienting effect at both at an SOA of 0 ms and 300 ms but, in addition, showed that this effect is higher at an SOA of 0 ms than at an SOA of 300 ms. In order to verify whether the gaze congruency index at a SOA of 0 ms was boosted by the increased attentional deployment onto the locus of the gazer, we compared its magnitude to the gaze congruency index found in Experiment 2 and in the original experiment conducted by Samson and colleagues (2010, Experiment 1). The gaze congruency index in the 0-ms SOA condition was significantly higher in this experiment than in Experiment 2, *t* (48) = 3.234, *p =*.002, *d* = 0.93, and in the original experiment conducted by Samson and colleagues (2010; Experiment 1), *t* (39) = 3.135, *p =* .007, *d* = 0.94. These results show that, due to the increased attentional deployment onto the location of the gazer, the attentional orienting effect was almost 3 times higher than the effect usually found in the classic version of the VPT paradigm (see Supplementary Figure 1).

------------------------------------------------

Insert Supplementary Figure 1 about here

------------------------------------------------

**Analyses including the other-perspective trials**

The ANOVA 2 (Perspective instruction: self vs. other-perspective) x 2 (Congruency) x 2 (SOA) conducted on the IESs revealed a significant main effect of Congruency, *F* (1, 24) = 45.792, *MSE* = 6,549,920, *p* < .001, η_p_² = .656, with better performances on congruent perspectives trials than on incongruent perspectives trials, a main effect of Perspective, *F* (1, 25) = 14.193, *MSE* = 621,248, *p* = .001, η_p_² = .372, with participants being better at judging their own perspective than the avatar’s perspective, a significant Congruency x Perspective interaction effect, *F* (1, 24) = 20.964, *MSE* = 1,910,733, *p* < .001, η_p_² = .466, and a significant main effect of SOA, *F* (1, 24) = 3,241.585, *MS*E = 401,339, *p* < .001, η_p_² = .868, with worse performances in the 0-ms SOA condition than in the 300-ms SOA condition.

To explore the interaction between Congruency and Perspective, a series of paired t-tests tested the effect of Congruency on self- and other-perspective trials and the effect of Perspective on congruent and incongruent perspectives trials. Paired t-tests showed a significant Congruency effect on both self- and other-perspective trials, *t* (24) = 7.029, *p* < .001, *t* (24) = 5.940, *p* < .001, respectively, with better performances on congruent perspectives trials. There was a significant Perspective effect on congruent perspective trials, *t* (24) = 3.597, *p* = .001, with participants being better at judging the avatar’s perspective than their own perspective, and a significant the effect of Perspective on incongruent perspective trials, *t* (24) = 4.407, *p* < .001, with participants being better at judging their own perspective and the avatar’s perspective. These results are in line with the results reported in the original paradigm (Samson et al., 2010).

**Supplementary Figure captions**

**Supplementary Figure 1.** Overview of the gaze congruency indexes reported in the gaze cueing paradigm and the visual perspective-taking paradigm from Experiment 1, 2, 3, S4, and Samson and colleagues (2010, Experiment 1). SOA = stimulus onset asynchrony, IES = inverse efficiency score. Error bars indicate the 95% confidence intervals. ** = *p* < .01, *** = *p* < .001 (selected comparisons).
