# Supplementary figures and images for: From gaze cueing to perspective taking: Revisiting the claim that we automatically compute where or what other people are looking at
Source: Vis cogn. 2016 Jan 24;23(8):1020–42. doi: 10.1080/13506285.2015.1132804 (PMC4743615; doi:10.1080/13506285.2015.1132804)

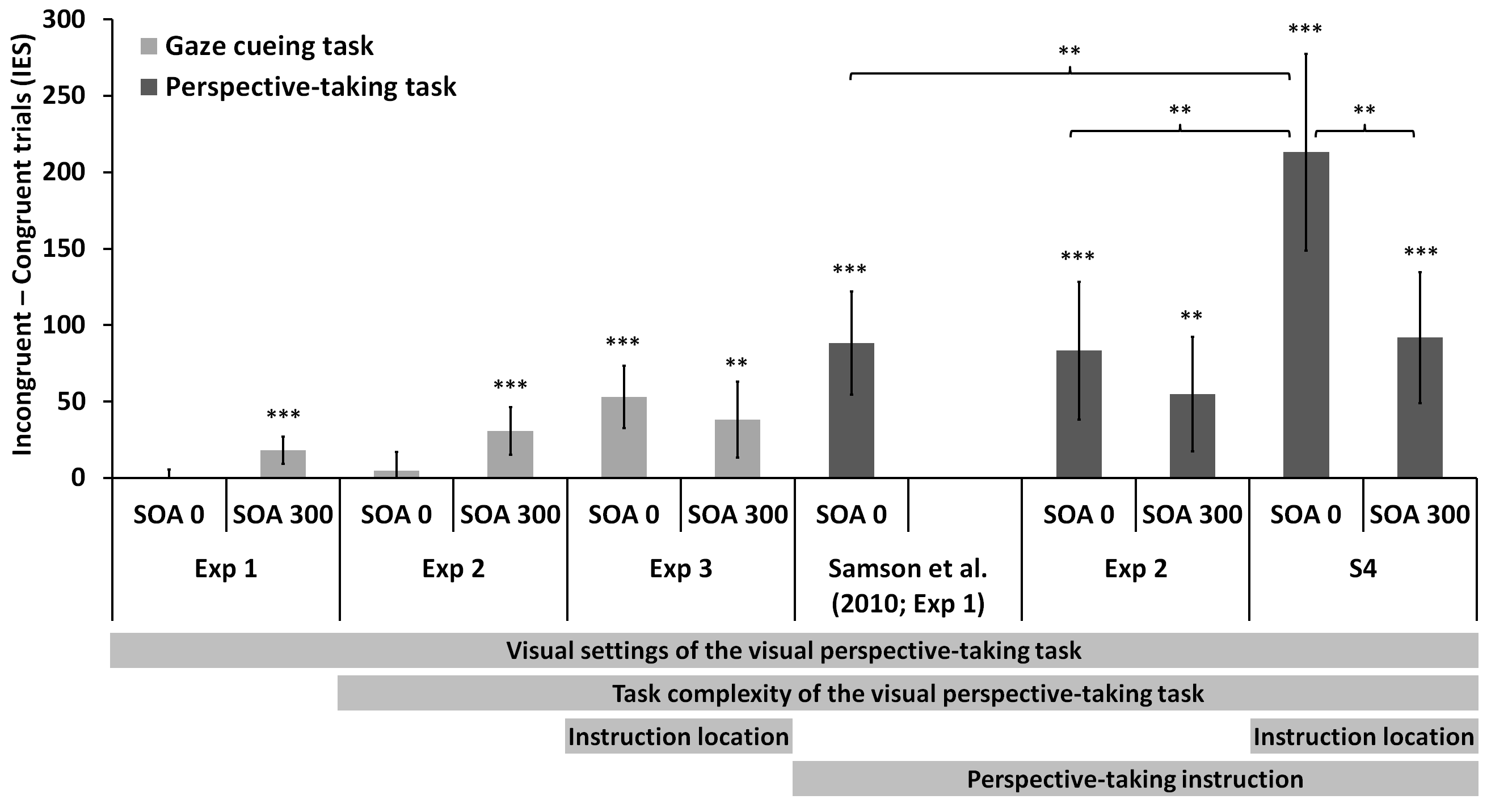

Supplement: Supplementary_Figure_1.tif [file pvis_a_1132804_sm0656.tif]
